# Death and Crime

**Published:** 1859-07

**Authors:** 


### DEATH AND CRIME.

Most of the charitable institutions of New York are filled with the poor and maimed, and sick. A large apartment of the city prison is crowded with drunken women, and three-fourths of the prisoners are mere boys; one half at least are under twenty-one years of age, and very few are ever reclaimed. When a boy (or girl) is once "put in jail," as the expression is, his *prestige* among his companions is lost; he feels mean before them; his *morale* is gone, and reckless desperation makes him lost for life.

Then there is a large class of older persons, who have no home beyond a cold and cheerless boarding place; others have no kindred, no associations, and out of the hours of active employment, do not know what to do with themselves. Not a few, for a variety of causes, are not happy at home. As to all these persons, time hangs heavy at night and on Sundays, and they lounge in public places, according to their *status;* some in the sitting rooms of the first hotels, down to the corner groggery, the theatre and the dance house; next the hospital, the poor house and then the potters' field.

We may mould the rising generation for a different destiny, but for the hopeless and homeless above fifteen, what shall we do? Hunt out good places for them? find work at good wages? That is not the speediest nor most practicable method of relief. We know very well that this is the general view of philanthropists, but men enough and money enough cannot be found to give every unoccupied male and female a plenty of work at even moderate wages, in any large city on the globe. Observant persons know very well, that the way to make a man of any body is to make him help himself; and that the inability to do that, oftener arises from the indisposition to do so, than from the impossibility of doing it. Show a man how, make him feel that he ought to take care of himself, and in nine cases out of ten he will go and do it. We must then appeal to the consciences of men, and we think the best way to do that is by what is called the "regularly appointed means of grace," by getting them to listen to the preaching of the gospel, to the reading of the scriptures, to the voice of prayer, and the songs of praise in public worship.

We believe the money spent on the vicious, and the sick poor of this city in one year, would build enough places of public worship to accommodate the whole of them. Each church should be plain, airy, with two large doors for exit in each wall, and opening outward. Each building should be large enough to have five thousand seats, rented at a dollar a year, in advance. Each pew should consist of three arm chairs, side by side, making each seat easily accessible, and preventing the loss of room by the thoughtless; nine out of ten will take the seat next the aisle, and make a barricade against the six, eight, or ten seats the other side of them. These five thousand seats would yield a revenue sufficient to keep the building in order, pay a sexton, and amply sustain the minister.

The very fact of a person owning the seat he occupies gives him a *status* in his own estimation, which of itself is elevating, encouraging and energizing.

Next, let it be arranged, that at proper times, the people be called on to deposite something for the general term of "benevolence," as they pass out of the house, after service. The very thought of a man, that he has done something for the poor and distressed, humanizes his own heart, makes him feel that there are others beneath him, increases his self-respect and appreciation, and he becomes by degrees familiarized with heaven born charity, loves it and performs it as a matter of course; and thus he becomes liberal without an effort, and that is true charity—that is a high bred generosity—it is being a Samaritan from principle, and he is saved for all time. For with self-respect and self-appreciation, there soon follow, as in a train, order, system, cleanliness, forethought and industry; while lovingness towards those still lower, crowns the whole.

But how get them to go to church in the first place? Four words tell the whole story, not original with us, "go," and say "come." "Go ye into the highways and hedges, and compel them to come in." Must you get on a stump or store box, and with Bible in hand, "preach"? No! You would not thus reach one in ten. But go up to a man, and cheerfully and courteously invite him to church. Do it aside, without any show of shame or secrecy. It is perfectly amazing how easily a man may be led to do even a disagreeable thing when approached in the right way. Get such an one to come once, but do not lose sight of him, encourage him to come again, by calling round to his place of occupation and saying a cheerful, respectful word or two about matters and things in general, and before you know it he will be interested, and will have his dollar for his "pew rent."

This is not all imagination and moonshine, manufactured in a doctor's office, on a bright spring morning, for this very moment we look out on a most dreary, drizzly, cold, raw February afternoon, everybody and everything dull and heavy as lead, except ourself.

There are difficulties in the way of all this. To be sure there are. Nothing of any account was ever accomplished without difficulty. But here is the master's command, "go" —"say come"—"compel them to come in." These words mean just what they say, and in a connection similar to the one under consideration.

"The great awakening" has swept across two continents; there is reason to believe that it originated in a line of conduct such as has been recommended, opportunely favored perhaps by the influences of the commercial disasters of "fifty seven." In the spring of that year we were in an upper room in Fifth Avenue, with some four hundred others, listening to a gentleman who was engaged in enlisting the churches to personal specific effort to induce a more general attendance on religious worship. The plan of operation was in effect as follows: to divide a certain area around a church building into districts, making, say, the four sides of a square or block of buildings one district, every dwelling in which was to be visited by a person volunteering such labor, with a view to ask each resident personally, to attend some religious services on Sundays, if in no regular attendance anywhere. The plan had been adopted in a number of churches, and had workea well. Two or three things were developed worthy of mention: a rebuff was of very rare occurrence and hurt nobody.

Persons were found living in some of the most elegant houses, and in splendid style ; and in one case there had been a five year's residence without ever having attended church. The occupant, a stranger in a strange city, was affected to tears on learning that some one in a million felt interest enough to give the invitation to go to the house of God.

A minister rose in his pulpit one day and said, "I see the representatives of near forty (we believe it was forty-seven) families here to day, who were never observed here before." It was the result of this district invitation. This system of things, this gentleman, whose name we do not remember, was engaged in carrying out all over New York, Brooklyn and other suburbs, and who shall say that this was not the leaven that began to diffuse its influences through the community, commencing in September following. Bring people into the sanctuary, let the gospel be dispensed to them, and the Almighty Spirit will take care of the rest; and we believe that, as to similar efforts elsewhere, or kept up here, it may be written up in due time "so shall my word be that goeth forth out of my mouth : it shall not return unto me void, but it shall accomplish that which I please, and it shall prosper in the thing whereto I sent it."

When it is taken into account that strangers are constantly moving into large cities, such a district visitation twice a year might do good ; and once a year as to all other churches in town and country. Very likely there is not a house of worship in the land, which has not within a mile of it, some person or family that has never been invited, courteously, kindly, considerately, to come in. Nobody felt any interest in them, the consciousness of which has hurt the feelings and wounded the spirit of many a worthy, but too retiring person. Surely one of the great wants of the age is a sincere, kindly, unobtrusive interest in those around us, not in our own immediate connection.

What we have written is proposed, not so much as a model, but as a neucleus about which thought and action may centre with a large libertyfor modification, according to greatly varying circumstances. Two things should however pervade every plan.

First—personal solicitation to attend religious worship.

Second—rigid advance payment of a small sum annually for each seat.

There are many considerations as to the good policy of this last, which will suggest themselves to those who best understand the human heart. Ownership elevates. Even casual association with well-dressed and cleanly persons elevates. The consciousness of a right above another, elevates. If a man gets into the habit of going to church, he after a while begins to feel "put out" when he does not attend. A man can hear better and more satisfactorily, when he occupies the same seat all the time, and if by any means he loses it, he is incommoded. If a man owns a seat for a year by purchase, there is more or less of the feeling "I'll get the worth of my money out of it any how." Besides, one is very apt to "strike up" a "speaking acquaintance" with the person next to whom he sits from time to time, and as the circle of any one's acquaintance extends with orderly, quiet persons, or those of substance and position, there are restraints upon his conduct, he is put more and more on his good behavior; and all this, while there are the dewy influences always falling, of the prayers and songs and word of grace which are peculiar to the house of God. We trust that the religious press will take up this subject, having a care not to spend time in arguing with people who talk about "laying a tax on religion," "freely ye have received, freely give." Address only those who believe the Bible, who believe that the world is to be regenerated by human agencies, by an active, aggressive christianity, which as much requires money, as a national warfare against invading foes, as shadowed forth in the Master's injunction in his farewell discourse—"He that hath a purse let him take it, and likewise his scrip, and he that hath no sword (no means;—Ed.) let him sell his garment and buy one."

